# Prospective study of intratumoral microvessel density, p53 expression and survival in colorectal cancer

**DOI:** 10.1038/sj.bjc.6690051

**Published:** 1999-01

**Authors:** P B Vermeulen, G G Van den Eynden, P Huget, G Goovaerts, J Weyler, F Lardon, E Van Marck, G Hubens, L Y Dirix

**Affiliations:** Angiogenesis Group, Oncological Centre; Department of Pathology, St. Augustinus Ziekenhuis, University of Antwerp (UIA), Wilrijk, B2610, Belgium; Department of Epidemiology and Community Medicine, University of Antwerp (UIA), Wilrijk, B2610, Belgium; Laboratory of Experimental Oncology, University of Antwerp (UIA), Wilrijk, B2610, Belgium; Department of Pathology, University of Antwerp (UIA), Wilrijk, B2610, Belgium; Department of Surgery, University of Antwerp (UIA), Wilrijk, B2610, Belgium

**Keywords:** colon cancer, angiogenesis, prognosis, morphometry

## Abstract

Adjuvant treatment of patients with colorectal cancer is hampered by a lack of reliable prognostic factors in addition to the clinicopathological staging system. A poorly defined but considerable fraction of Astler–Coller stage B patients will experience tumour recurrence, and some of the stage C patients will probably survive for a prolonged time after surgery without adjuvant treatment. Assessing parameters related to tumour angiogenesis has provided valuable prognostic information in different tumour types. The formation of new microvessels is part of the malignant phenotype in the majority of tumours. Alterations in tumour-suppressor genes, such as the *p53* gene, or oncogenes, such as the *ras* gene, have been found to be responsible for changing the local balance of pro- and antiangiogenic factors in favour of the former. In this prospective study, intratumoral microvessel density (IMD) was assessed by immunostaining tissue sections for CD31 and counting individual microvessels in selected and highly vascular regions in specimens of 145 colorectal cancer patients. p53 protein overexpression was semiquantitatively determined after immunohistochemistry. In both uni- and multivariate analysis, high IMD was significantly associated with shorter survival in the patients undergoing surgery with curative intent (Astler–Coller stages A–C). p53 added prognostic power to IMD, both in Astler–Coller stage B and stage C patients. An association between IMD and mode of metastasis was also noted. High IMD was strongly associated with the incidence of haematogenous metastasis during follow-up, but not with the presence of lymphogenic metastasis observed at surgery. This study confirms the results of previous retrospective analyses of IMD and survival in colorectal cancer and warrants a clinical validation by randomizing stage B tumour patients with high IMD and p53 overexpression between adjuvant treatment or not. © 1999 Cancer Research Campaign


*British Journal of Cancer* (1999) **79**(2), 316–322
© 1999 Cancer Research Campaign

### Prospective study of intratumoral microvessel density, p53 expression and survival in colorectal cancer

**PB Vermeulen[1], GG Van den Eynden[1], P Huget[1], G Goovaerts[2], J Weyler[3], F Lardon[4], E Van Marck[5], G Hubens[6] and LY Dirix[1]**

[1]Angiogenesis Group, Oncological Centre and [2]Department of Pathology, St. Augustinus Ziekenhuis, and [3]Department of Epidemiology and Community Medicine, [4]Laboratory of Experimental Oncology, and [5]Department of Pathology, [6]Department of Surgery, University of Antwerp (UIA), B2610 Wilrijk, Belgium.

**Summary** Adjuvant treatment of patients with colorectal cancer is hampered by a lack of reliable prognostic factors in addition to the clinicopathological staging system. A poorly defined but considerable fraction of Astler–Coller stage B patients will experience tumour recurrence, and some of the stage C patients will probably survive for a prolonged time after surgery without adjuvant treatment. Assessing parameters related to tumour angiogenesis has provided valuable prognostic information in different tumour types. The formation of new microvessels is part of the malignant phenotype in the majority of tumours. Alterations in tumour-suppressor genes, such as the *p53* gene, or oncogenes, such as the *ras* gene, have been found to be responsible for changing the local balance of pro- and antiangiogenic factors in favour of the former. In this prospective study, intratumoral microvessel density (IMD) was assessed by immunostaining tissue sections for CD31 and counting individual microvessels in selected and highly vascular regions in specimens of 145 colorectal cancer patients. p53 protein overexpression was semiquantitatively determined after immunohistochemistry. In both uni- and multivariate analysis, high IMD was significantly associated with shorter survival in the patients undergoing surgery with curative intent (Astler–Coller stages A–C). p53 added prognostic power to IMD, both in Astler–Coller stage B and stage C patients. An association between IMD and mode of metastasis was also noted. High IMD was strongly associated with the incidence of haematogenous metastasis during follow-up, but not with the presence of lymphogenic metastasis observed at surgery. This study confirms the results of previous retrospective analyses of IMD and survival in colorectal cancer and warrants a clinical validation by randomizing stage B tumour patients with high IMD and p53 overexpression between adjuvant treatment or not.

**Keywords:** colon cancer; angiogenesis; prognosis; morphometry

In colorectal cancer, one of the most common malignant tumours, factors predicting relapse risk in addition to the currently used clinicopathological staging systems are needed for several reasons. A considerable fraction of node-negative colorectal tumours will recur (Olson et al, 1980) and adjuvant therapy is not routinely administered for this group (Moertel et al, 1990). Also positive lymph nodes do not always clearly predict a more unfavourable outcome (Wolmark et al, 1986). The Dukes' system has, therefore, been extended by subdividing each stage according to depth of invasion (American Joint Committee on Cancer, 1988). It is, however, conceivable that because of inadequate sampling both lymph node status and depth of mural penetration are inaccurately assessed. The identification and adjuvant treatment of those stage B and C tumour patients prone to early relapse might improve the survival of colorectal cancer patients without the risk of overtreatment.

Angiogenesis is necessary for tumour growth (Folkman et al, 1990) and facilitates two processes responsible for the malignant phenotype of a growing tumour, i.e. invasion and metastasis (Liotta et al, 1974; Skobe et al, 1997). Tumour growth kinetics correlate with the concentration of angiogenic factors in the blood of advanced colorectal cancer patients (Dirix et al, 1996, 1997). Although the proliferation fraction of a tumour does not depend on the number of microvessels, tumour cells are less prone to undergo apoptosis in well-vascularized tumours (Holmgren et al, 1995; Lu et al, 1997). Invasion of several in situ carcinoma types can be predicted by elevated microvessel counts at the other side of the basement membrane (Hanahan et al, 1996). Quantification of the density of the vasculature in selected areas of the tumour can reproducibly predict metastasis, provided a standardized method is used (Vermeulen et al, 1996*a*). Although in different animal tumour models the angiogenic phenotype or 'angiogenic switch' is related to accelerated growth and metastasis, angiogenesis-independent growth exists (Pezzella et al, 1997).

The prognostic significance of angiogenesis in patients with colorectal cancer has been reported, but none of the studies had a prospective design (Saclarides et al, 1994; Frank et al, 1995; Takahashi et al, 1995, 1997; Tomisaki et al, 1996; Engel et al, 1996; Takebayashi et al, 1996*a*, 1996*b*; Tanigawa et al, 1997). Most of the few negative studies used a counting technique which substantially differs from the international consensus method of intratumoral microvessel density (IMD) assessment (Bossi et al, 1995; Lindmark et al, 1996). We have previously confirmed the reproducibility of this method in colorectal and breast cancer specimens (Vermeulen et al, 1995*a*, 1997).

Several genes, like *p53* and *ras*, might be responsible for the angiogenic switch by changing the local balance between pro- and antiangiogenic factors (Rak et al, 1995). *p53* probably regulates

*Received 29 January 1998*
*Revised 27 April 1998*
*Accepted 29 April 1998*

*Correspondence to:* LY Dirix, Angiogenesis Group – Experimental Oncology, Oncological Centre, A.Z. St.-Camillus/St.-Augustinus, Oosterveldlaan 24, B2610 Wilrijk-Antwerp, Belgium the thrombospondin 1 (TSP-1) gene in a direct way (Dameron et al, 1994). The secretion of TSP-1, a potent angiogenesis inhibitor, is reduced in fibroblasts isolated from Li–Fraumeni patients because of the in vitro loss of the remaining wild-type *p53* allele. Transfection of human embryonic kidney cells, having a low level of endogenous p53 protein, with a dominant mutant *p53* gene resulted in increased production of vascular endothelial growth factor (VEGF) mRNA (Kieser et al, 1994). The mechanism by which p53 regulates angiogenesis appears to be tissue specific because in glioblastoma an angiogenesis inhibitory activity different from TSP-1 was controlled by the wild-type *p53* gene (Van Meir et al, 1994).

In this prospective study, to determine whether the extent of the tumour vasculature would be a predictor of patient's survival and of the mode of metastasis, we compared the clinical data of 145 patients with colorectal cancer with IMD and with *p53* expression status of their primary tumour specimens.

### PATIENTS AND METHODS

### Patients

For this study, clinical data and tumour tissue from 170 patients were collected between January 1991 and July 1995 at the departments of surgery of the St. Augustin General Hospital and of the University Hospital, Antwerp, Belgium. Patients with synchronous or metachronous tumours were excluded ($n$ = 11), as were patients who did not experience a disease-specific death ($n$ = 8). Six patients with incomplete follow-up data were also omitted, resulting in 145 patients suitable for analysis.

Tumours were evaluated according to the modified Astler–Coller clinicopathological classification. Patients did not receive chemotherapy or radiation therapy before surgery. All had primary tumour resection with regional lymph node dissection. Tumour specimens were fixed in buffered formalin solution and embedded in paraffin. Histological grading was performed on haematoxylin and eosin (H and E)-stained sections. Standardized adjuvant treatment was delivered to all stage C patients, with the 5-fluorouracil and levamisole regimen (Moertel et al, 1990). Survival was measured from the time of resection until death or the end of July 1997, whichever came first. Haematogenous metastasis was evaluated both at surgery and during standardized follow-up, whereas lymphogenic metastasis was histologically confirmed only at surgery. Prognostic information on all patients was transferred to a dedicated computer file.

### Immunohistochemical study for CD31 and p53 antigens

The staining methods have been described before (Vermeulen et al, 1996*b*). Briefly, 5-μm-thick sections, mounted on 3-aminopropyltriethoxysilane-coated slides, were dewaxed in xylene, rehydrated in ethanol and incubated in 0.3% hydrogen peroxide in methanol for 30 min. The sections were pretreated in the microwave oven in a citrate buffer for 15 min at 95°C and left to cool for 1 h at room temperature. Blood vessels were visualized by staining endothelial cells for CD31 (mouse monoclonal antibody JC70, Dako, Prosan, Belgium). On a parallel section, immuno staining for the p53 protein was performed by applying the mouse monoclonal antibody DO7 (Biogenex, Klinipath, Belgium), recognizing both the mutant and the wild-type p53 protein. A standard double peroxidase/antiperoxidase technique was used with diaminobenzidine tetrahydrochloride as chromogen. A light H and E counterstain was applied. Negative controls consisted of omission of the primary antisera.

### Determination of intratumoural microvessel density

IMD was assessed according to the international consensus report (Vermeulen et al, 1996*a*). One section per tumour was analysed. The entire section was systematically scanned at × 100 magnification to identify the areas of most intense neovascularization, commonly called 'hot spots'. Those were identified as having the highest density of brown-staining, CD31-positive cells or cell clusters. Vascular hotspots were suitable for analysis provided they were adjacent to tumour cells. Whenever such a highly vascularized area was encountered at × 100 magnification, individual microvessels were counted in a single × 200 field (0.61 $mm^2$) in this area. Any brown-staining structure clearly separated from adjacent microvessels was regarded as a single, countable microvessel. Neither vessel lumens nor the presence of red blood cells were used to define a microvessel, and no exclusion criteria based on size were used. Occasional immunopositive macrophages and plasma cells were excluded on morphological grounds. After vessels in the × 200 field were counted, scanning of the tumour section at × 100 magnification was continued until analysis of the entire section. The mean of the five highest counts per tumour was taken for further analyses and was expressed as microvessels per field at magnification × 200. The predefined cut-off value for categorical evaluation of IMD was the median IMD of the population studied.

### Determination of p53 expression p53 expression was graded semiquantitatively. If more than 10% of all tumour cell nuclei were stained, the tumour was regarded as positive. This cut-off value was also predefined at the start of the study.

### Statistical analysis

The clinical characteristics of the patients in relation to IMD were compared by Mann–Whitney $U$-test and $\chi^2$ test. The survival curves were plotted according to the Kaplan–Meier method and differences were validated by Mantel–Cox logrank testing. The influence of various factors on overall survival was assessed by multiple regression by the Cox proportional hazards model. For all analyses, the Statview 4.0 application (Abacus Concepts, Berkeley, CA, USA) was used. A value of $P < 0.05$ was considered statistically significant.

### RESULTS

### Patients

Median age at diagnosis was 66 years (range 25–88). Median follow-up time was 30 months (range 2–77). Median follow-up of the surviving patients was 43 months (range 24–77). Half of the patients had died after 59.7 months (s.e. 14.5). The male/female ratio was 1.08. Five patients (4%) had stage A disease, 44 (30%) had stage B disease (three $B_1$, 35 $B_2$ and six $B_3$), 67 (46%) had stage C disease (three $C_1$, 54 $C_2$, ten $C_3$), and 29 (20%) had stage D disease. In 112 patients (77%), the tumour was located in the left

© Cancer Research Campaign 1999

colon or rectum. In 33 patients (23%), the tumour was located in the right or transverse colon. One quarter of all patients had a rectal tumour. Median tumour size was 40 mm (range 15–120). Astler–Coller stage was significantly associated with survival ($P$ < 0.0001). None of the stage A patients died during the study. Survival of stage B and stage C patients was comparable, with respective 5-year survival rates of 65% [95% confidence interval (95% CI) 45–85] and 58% (95% CI 43–73). Survival of stage $C_3$ patients was inferior to the survival of other stage C patients ($P$ = 0.0002). The overall survival rate of 5 years of this group was 15% (95% CI 0–40) and the overall survival rate of 3 years was 30% (95% CI 2–58). All patients with stage D tumours had died within 3 years after surgery.

### Intratumoral microvessel density and overall survival

Median IMD of all patients was 74 (range 39–188). Median IMD of patients undergoing curative surgery (stages A–C) was 75 (range 39–188; $n$ = 116). IMD was not related to Astler–Coller stage ($P$ > 0.1). The median IMD of tumours of patients younger than 66 years was significantly higher than the median IMD of tumours of older patients: 83 compared with 66 respectively ($P$ = 0.035). Linear regression analysis of IMD vs age resulted in a coefficient $r$ of –0.2 ($P$ = 0.037).

When regarded as a continuous variable, a trend of increasing IMD being related to shorter survival in patients undergoing surgery with curative intent was observed [hazard ratio expressed per increment of one microvessel = 1.01 (95% CI 1.00–1.02), $P$ = 0.08]. In Astler–Coller stage D patients, there was no relation between IMD and survival.

The 116 patients with stages A–C tumours were dichotomized by the median IMD of 75 into two subgroups: 57 patients with hypovascular tumours and 59 patients with hypervascular tumours. The $\chi^2$ analysis demonstrated that tumour site, tumour size, differentiation grade, depth of penetration, p53 protein expression status and the presence or absence of lymph node metastasis were equally distributed among the two subgroups (Table 1). Younger patients and tumours with more than three metastastic lymph nodes were more frequent in the hypervascular subgroup, although this was only a statistical trend.

Haematogenous metastasis was significantly more frequent in the hypervascular subgroup ($P$ = 0.001) (Table 1). The survival rates of the two subgroups calculated using the Kaplan–Meier method were significantly different in favour of the hypovascular group ($P$ = 0.005; Figure 1). Although 72% (95% CI 52–92) of the hypovascular group reached the overall survival rate of 5 years, only 51% (95% CI 37–66) of the hypervascular group survived for

**Table 1** Comparison of clinicopathological features between patients undergoing curative surgery with hypovascular and those with hypervascular tumours

| Variable | Hypovascular IMD < 75 ($n$ = 57) | Hypervascular IMD ≥ 75 ($n$ = 59) | $P$-value |
|---|---|---|---|
| Age[a] | | | |
| < 66 | 24 (41) | 35 (59) | 0.06 |
| ≥ 66 | 33 (58) | 24 (42) | |
| Tumour site | | | |
| Left, rectum | 45 (48) | 48 (52) | NS |
| Right transverse | 12 (52) | 11 (48) | |
| Tumour size[b] | | | |
| < 4 cm | 28 (42) | 38 (58) | NS |
| ≥ 4 cm | 29 (58) | 21 (42) | |
| Tumour histology | | | |
| Well–mod. differentiated | 50 (49) | 53 (51) | NS |
| Poorly differentiated | 7 (54) | 6 (46) | |
| Depth of penetration[c] | | | |
| T1, T2 | 6 (60) | 4 (40) | NS |
| T3, T4 | 51 (48) | 55 (52) | |
| p53 expression | | | |
| – | 20 (42) | 27 (58) | NS |
| + | 37 (54) | 32 (46) | |
| Lymph node metastasis[d] | | | |
| Negative | 27 (56) | 21 (44) | NS |
| Positive | 30 (45) | 37 (55) | |
| ≤ 3 | 50 (54) | 43 (46) | 0.06 |
| > 3 | 7 (32) | 15 (68) | |
| Haematogenous metastasis | | | |
| Negative | 48 (59) | 34 (41) | 0.001 |
| Positive | 9 (26) | 25 (74) | |

[a],[b]Cut-off value is the median value; [c]T1, penetrates to submucosa; T2, penetrates to subserosa; T3, serosa (exposed); T4, infiltrating the neighbouring tissue; [d]evaluated at surgery.

© Cancer Research Campaign 1999

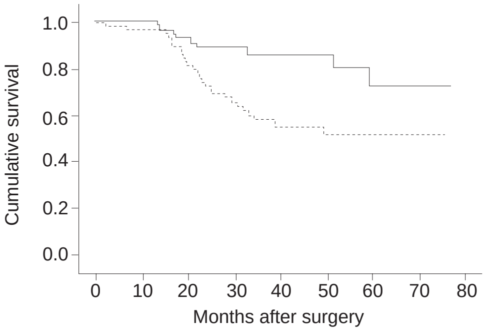

**Figure 1** Overall survival curve for patients with stages A–C tumours. Survival of 57 patients with hypovascular tumours [intratumoral microvessel density (IMD) < 75, solid line) was significantly greater than that of the 59 patients with hypervascular tumours (IMD ≥ 75, dashed line). $P = 0.0047$ by logrank (Mantel–Cox) test

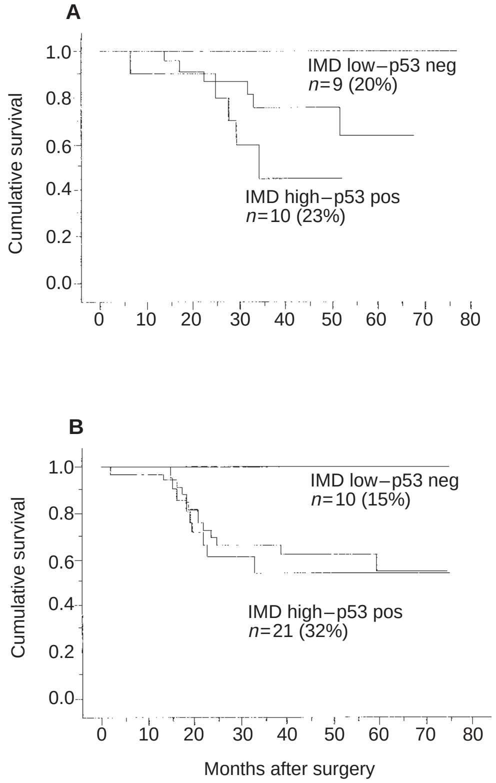

**Figure 2** (**A**) Overall survival curve for patients with stage B tumours according to intratumoral microvessel density (IMD) and p53 overexpression. Patients with tumours characterized by IMD < 75 (IMD low) and no p53 expression (p53 neg) had better survival than patients with IMD ≥ 75 (IMD high) and p53 expression (p53 pos) [$P < 0.03$ by logrank (Mantel–Cox) test]. (**B**) The same survival analysis as for stage B tumours was performed for stage C tumours that period. An IMD of 75 or more yielded a relative risk of cancer death of 2.85 (95% CI 1.33–6.09). A high IMD was associated with inferior survival also when tertiles were used as cutoff values. The respective overall survival rates of 5 years for high, intermediate and low IMD groups were 51% (95% CI 33–70), 56% (95% CI 34–77) and 76% (95% CI 54–99) ($P = 0.040$). In the 29 patients with stage D tumours, no association of IMD with survival was found.

Of the 116 patients with stages A–C tumours, 20 patients (17%) had a hypovascular tumour without p53 overexpression. The overall survival rate of 5 years was 100% in this group compared with 58% (95% CI 42–75) in the group of tumours being hypervascular or overexpressing p53, and 51% (95% CI 32–71) in the group of tumours being hypervascular and overexpressing p53 ($P < 0.01$). There was no difference in the distribution of A–C stages in the three groups ($P > 0.1$). The same survival analysis was performed for stage B and stage C tumour patients separately (Figure 2A and B). Of all stage B tumour patients, 23% had an unfavourable prognosis and 20% had an extremely favourable prognosis. A decision to treat or not might, thus, be facilitated in 43% of all stage B tumour patients. Of all stage C patients, 15% might not need adjuvant treatment on the basis of IMD and p53 expression. There was no difference in the distribution of substages according to depth of invasion in the different p53–IMD groups ($P > 0.1$).

The prognostic value of IMD in patients undergoing curative surgery was examined by multivariate analysis using the Cox proportional hazards model (Table 2). IMD was identified as a significant and independent prognostic factor. Tumour size attained borderline significant prognostic value. A statistical trend towards prognostic significance was reached for differentiation grade, p53 protein expression status and age. When, in a second model, lymph node metastasis was expressed as more or less than three nodes involved, this was also not associated with survival. Size now became a significant prognostic factor. All other *P*-values did not change.

### Intratumoral microvessel density and mode of metastasis

Of the 116 stages A–C tumour patients, 34 (29%) developed haematogenous metastasis after surgery. Median IMD in this

**Table 2** Association of various factors with overall survival determined by the Cox proportional hazards model for patients undergoing curative surgery

| Prognostic factors | Hazards ratio | 95% Confidence limits | *P*-value |
|---|---|---|---|
| Age (< 66 vs. ≥ 66)[a] | 1.93 | 0.89–4.20 | 0.098 |
| Tumour site (left, rectum vs. right, transverse) | 1.58 | 0.48–5.16 | 0.453 |
| Tumour size (> 40 vs. ≤ 40 mm)[a] | 2.16 | 0.99–4.74 | 0.054 |
| Histology (poorly differentiated vs. other) | 3.45 | 0.90–13.25 | 0.072 |
| Tumour depth (T3,4 vs. T1,2) | 1.97 | 0.26–14.92 | 0.512 |
| Lymph node metastasis (+ vs. –) | 1.13 | 0.50–2.53 | 0.774 |
| p53 (+ vs. –) | 2.21 | 0.91–5.37 | 0.079 |
| IMD (≥ 75 vs. < 75)[a] | 3.26 | 1.41–7.55 | 0.006 |

[a]Cut-off value is the median value.

© Cancer Research Campaign 1999

group was higher than in the group of patients without haematogenous metastasis: 87 compared with 72 ($P$ = 0.004). There was no difference in IMD between patients with and without lymph node metastasis detected at surgery. The prevalence of haematogenous metastasis, but not of lymph node metastasis, increased as IMD increased per tertile ($P$ = 0.011) (Figure 3). Median IMD of stage D tumours was 69, and was significantly lower than the median IMD of stages A–C tumours of patients who developed haematogenous metastasis after surgery ($P$ = 0.023).

### DISCUSSION

This prospective study confirms the prognostic value of IMD in colorectal cancer patients previously suggested by retrospective studies, and demonstrates the additive prognostic value of IMD and p53. Analysis of the relation of IMD with mode of metastasis reveals a positive association of IMD with the incidence of haematogenous metastasis in colorectal cancer patients. All significant results are restricted to patients treated by surgery with curative intent, thus excluding Astler–Coller stage D tumour patients.

In eight retrospective studies, a significant correlation between high IMD and shorter survival in colorectal cancer was found by univariate analysis (Saclarides et al, 1994; Frank et al, 1995; Tomisaki et al, 1996; Engel et al, 1996; Takebayashi et al, 1996a, 1996*b*; Takahashi et al, 1997; Tanigawa et al, 1997). In five of these studies, a multivariate analysis was performed, by which in four out of the five studies the prognostic value of IMD was confirmed. In three reports, IMD in the primary tumour was positively correlated with respectively platelet-derived endothelial cell growth factor (PdECGF) expression (Takebayashi et al, 1996*b*), VEGF expression and p53 protein overexpression (Kang et al, 1997), and IMD in the liver metastases in stage D tumour patients (Mooteri et al, 1996). In univariate analysis, high PdECGF expression (Takebayashi et al, 1996*b*) and low IMD in the liver metastases (Mooteri et al, 1996) were associated with inferior survival. One study reported a lack of correlation between IMD and survival in colorectal cancer (Bossi et al, 1995) and another study yielded a significant association between high IMD and longer survival, both in uni- and multivariate analysis (Lindmark et al, 1996). Interestingly, the last two studies are based on a methodology which deviates considerably from the microvessel counting method originally introduced by Weidner et al (1991) and recently modified in an international consensus report (Vermeulen et al, 1996*a*).

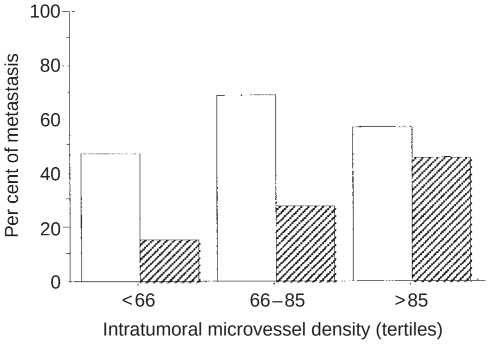

**Figure 3** Metastatic disease among 116 patients with stages A–C in relation to IMD in progressive tertile increments. The incidence of lymph node metastasis (□) does not change ($P$ > 0.1), whereas the prevalence of haematogenous metastasis (▨) increased as IMD increased ($P$ = 0.011)

Wild-type p53 expression has been shown in vitro to restrict the ability of cells to induce angiogenesis (for review: Bouck, 1996). In colorectal cancer, IMD has been reported to be higher in tumours characterized by the accumulation of an afunctional p53 protein, as detected by immunohistochemistry (Vermeulen et al, 1996*b*; Kang et al, 1997). Although we have not been able to confirm this, patients with tumours without p53 protein accumulation combined with a low IMD had a significantly better survival than other patients. This additive prognostic power of IMD and p53 status has been reported in node-negative breast carcinoma (Gasparini et al, 1994). It might be that mutant p53 is responsible for the secretion of factors which induce remodelling of the tumour vasculature. This might lead, in highly vascularized tumours, to more extensive mitogenic paracrine signalling towards malignant cells than in less vascularized tumours (Folkman, 1996). In contrast, high IMD might be the reflection of other genes besides *p53* residing in a state in which they both augment angiogenesis and accelerate tumour growth, like the mutated *ras* gene (Rak et al, 1995).

The incidence of distant relapse was higher in well-vascularized tumours. This observation supports the theoretical basis of counting microvessels in areas which are most densely vascularized. Tumour cells are expected to intravasate more easily when surrounded by a larger surface area of endothelial cells or by more newly formed vascular sprouts. Proliferating and migrating endothelial cells digest the extracellular matrix by different proteases (Brooks et al, 1996), and stimulate tumour cell activity by paracine pathways (Folkman, 1996). We have observed a two-fold higher endothelial cell proliferation rate in vascular hotspots compared with unselected regions in colorectal cancer specimens (Vermeulen et al, 1995*b*). In breast cancer, tumour cell shedding during surgery was positively correlated with the number of microvessels per surface area in the vascular hotspots (McCulloch et al, 1995). In bone marrow aspirates from breast cancer patients before surgery, the likelihood of detecting micrometastases was positively associated with tumour vascularity and with vascular invasion (Fox et al, 1997).

Although the prevalence of lymph node metastasis at surgery was not associated with IMD in the primary tumour, patients with more than three involved lymph nodes more frequently had hypervascular tumours. Halting angiogenesis by antibodies blocking the VEGF receptors has recently been shown to suppress tumour cell invasion as well (Skobe et al, 1997). This is in direct support of the concept that endothelial or other stromal cells are responsible for at least part of the malignant phenotype of tumour cells, and might explain the observed relation between lymphogenic metastasis and IMD. In addition, tumour cell clones trapped in lymph nodes might grow more easily to a detectable size if they elicit more microvessels. The angiogenic phenotype of the metastatic cells probably reflects the angiogenic capacity of the primary tumour.

An explanation for the lack of association between IMD and survival in stage D tumour patients is not obvious. Survival of patients after resection of these tumours might at least be partly influenced by IMD in the metastasis. Low IMD in liver metastasis of 32 stage D patients has been found to predict shorter survival (Mooteri et al, 1996). IMD of the metastases reflected IMD of the primary colorectal tumours. A hypothetical explanation for the lack of association between IMD and survival is based on the highly invasive nature of stage D tumours. It is conceivable that tumour cells with these properties can migrate and proliferate in the stromal tissue of highly vascularized target organs without the

*© Cancer Research Campaign 1999*

need to elicit microvessel development. Such an angiogenesis-independent growth pattern has been observed in tumours in the lungs and in the liver (McGuire et al, 1997; Pezzella et al, 1997). In this way, the angiogenic properties of the primary tumour, reflected in the IMD value, would not necessarily be related to the potential of metastases to expand. A second explanation might be that Astler–Coller or Dukes' stage is not simply determined by the duration between initiation and diagnosis of colorectal cancer. Our observation of a significant difference between IMD of stage D tumours and IMD of stages A–C tumours of patients who developed metastases during follow-up supports this view. Moerkerk et al (1994) have shown that both the number and the type of Ki-*ras* oncogene mutations are related to Dukes' stage.

The observation of decreasing IMD with increasing age is supported by several animal experiments, and is in accordance with slower tumour growth rates and less frequent metastasis in old animals under experimental conditions (Ershler et al, 1997).

As the selection of colorectal cancer patients likely to benefit from adjuvant chemotherapy remains difficult, the clinical validation of the results of this prospective study seems warranted. Based on IMD and p53 status, a selection of stage B tumour patients prone to early relapse should be randomized between adjuvant treatment or not. Other prognostic indicators, such as chromosome 18q loss of heterozygosity, may be compared with IMD and p53 expression to identify stage B tumour patients at high risk for recurrence (Ogunbiyi et al, 1998). In the same way, a fraction of stage C tumour patients with reduced risk for recurrent cancer can be excluded from further treatment.

### ACKNOWLEDGEMENT

E Van Marck was supported by a grant of the FWO (Fonds Wetenschappelijk Onderzoek) Flanders, grant no. 1.5.234.97N.

### REFERENCES

American Joint Committee on Cancer (1988) Colon and rectum. In *Manual for Staging of Cancer*. Beahrs O, Henson D and Hutter R (eds.), pp. 75–80. Lippincott: Philadelphia

Bossi P, Viale G, Lee AKC, Alfano R, Coggi G and Bosari S (1995) Angiogenesis in colorectal tumors: microvessel quantification in adenomas and carcinomas with clinicopathological correlations. *Cancer Res* **55**: 5049–5053

Bouck N (1996) p53 and angiogenesis. *Biochim Biophys Acta* **1287**: 63–66

Brooks PC, Strömblad S, Sanders LC, von Schalscha TL, Aimes RT, Stetler-Stevenson WG, Quigley JP and Cheresh DA (1996) Localization of matrix metalloproteinase MMP-2 to the surface of invasive cells by interaction with integrin αvβ3. *Cell* **85**: 683–693

Dameron KM, Volpert OV, Tainsky MA and Bouck N (1994) Control of angiogenesis in fibroblasts by p53 regulation of thrombospondin-1. *Science* **265**: 1582–1584

Dirix LY, Vermeulen PB, Hubens G, Benoy I, Martin M, De Pooter C and Van Oosterom AT (1996) Serum fibroblast growth factor and tumour growth kinetics in advanced colorectal cancer. *Ann Oncol* **7**: 843–848

Dirix LY, Vermeulen PB, Pawinski A, Prové A, Benoy I, De Pooter C, Martin M and Van Oosterom (1997) Elevated levels of the angiogenic cytokines basic fibroblast growth factor and vascular endothelial growth factor in sera of cancer patients. *Br J Cancer* **76**: 238–243

Engel J, Bennett ST, Chambers AF, Doig GS, Kerkvliet N and O'Malley FP (1996) Tumor angiogenesis predicts recurrence in invasive colorectal cancer when controlled for Dukes' staging. *Ann J Surg Pathol* **20**: 1260–1265

Ershler WB and Longo DL (1997) Aging and cancer: issues of basic and clinical science. *J Natl Cancer Inst* **89**: 1489–1497

Folkman J (1990) What is the evidence that tumors are angiogenesis dependent? *J Natl Cancer Inst* **82**: 4–6

Folkman J (1996) Tumor angiogenesis and tissue factor. *Nature Med* **2**: 167–168

Fox SB, Leek RD, Bliss J, Mansi JL, Gusterson B, Gatter KC and Harris AL (1997) Association of tumor angiogenesis with bone marrow micrometastases in breast cancer patients. *J Natl Cancer Inst* **89**: 1044–1049

Frank RE, Saclarides TJ, Leurgans S, Speziale NJ, Drab EA and Rubin DB (1995) Tumor angiogenesis as a predictor of recurrence and survival in patients with node-negative colon cancer. *Ann Surg* **222**: 695–699

Gasparini G, Weidner N, Bevilacqua P, Maluta S, Dalla Palma P, Caffo O, Barbareschi M, Boracchi P, Marubini E and Pozza F (1994) Tumor microvessel density, p53 expression, tumor size, and peritumoral lymphatic vessel invasion are relevant prognostic markers in node-negative breast carcinoma. *J Clin Oncol* **12**: 454–466

Hanahan D, Christofori G, Naik P and Arbeit J (1996) Transgenic mouse models of tumour angiogenesis: the angiogenic switch, its molecular controls, and prospects for preclinical therapeutic models. *Eur J Cancer* **32A**: 2386–2393

Holmgren L, O'Reilly MS and Folkman J (1995) Dormancy of micrometastases: balanced proliferation and apoptosis in the presence of angiogenesis suppression. *Nature Med* **1**: 149–153

Kang S, Maeda K, Onoda N, Chung Y, Nakata B, Nishiguchi Y and Sowa M (1997) Combined analysis of p53 and vascular endothelial growth factor expression in colorectal carcinoma for determination of tumor vascularity and liver metastasis. *Int J Cancer* **74**: 502–507

Kieser A, Weich Ha, Brandner G, Marmé D and Kolch W (1994) Mutant p53 potentiates protein kinase C induction of vascular endothelial growth factor expression. *Oncogene* **9**: 963–969

Lindmark G, Gerdin B, Sundberg C, Påhlman L, Bergström R and Glimelius B (1996) Prognostic significance of the microvessel count in colorectal cancer. *J Clin Oncol* **14**: 461–466

Liotta LA and Saidel GM (1974) Quantitative relationship of intravascular tumor cells, tumor vessels, and pulmonary metastases following tumor implantation. *Cancer Res* **34**: 997–1004

Lu C and Tanigawa N (1997) Spontaneous apoptosis is inversely related to intratumoral microvessel density in gastric carcinoma. *Cancer Res* **57**: 221–224

McCulloch P, Choy A and Martin L (1995) Association between tumour angiogenesis and tumour cell shedding into effluent venous blood during breast cancer surgery. *Lancet* **346**: 1334–1335

McGuire BM, Cherwitz DL, Rabe KM and Ho SB (1997) Small-cell carcinoma of the lung manifesting as acute hepatic failure. *Mayo Clin Proc* **72**: 133–139

Moerkerk P, Arends J, Van Driel M, De Bruine A, De Goeij A and Ten Kate J (1994) Type and number of Ki-*ras* point mutations relate to stage of human colorectal cancer. *Cancer Res* **54**: 3376–3378

Moertel CG, Fleming TR, MacDonald JS, Haller DG, Laurie JA, Goodman PJ, Ungerleider JS, Emerson WA, Tormey DC, Glick JH, Veeder MH and Mailliard JA (1990) Levamisole and fluorouracil for adjuvant therapy of resected colon carcinoma. *N Engl J Med* **322**: 352–358

Mooteri S, Rubin D, Leurgans S, Jakate S, Drab E and Saclarides T (1996) Tumor angiogenesis in primary and metastatic colorectal cancers. *Dis Colon Rectum* **39**: 1073–1080

Ogunbiyi OA, Goodfellow PJ, Herfarth K, Gagliardi G, Swanson PE, Birnbaum EH, Read TE, Fleshman JW, Kodner IJ and Moley JF (1998) Confirmation that chromosome 18q allelic loss in colon cancer is a prognostic indicator. *J Clin Oncol* **16**: 427–433

Olson RM, Perencevich NP, Malcolm AW, Chaffey JT and Wilson RE (1980) Patterns of recurrence following curative resection of adenocarcinoma of the colon and rectum. *Cancer* **45**: 2969–2974

Pezzella F, Pastorino U, Tagliabue E, Andreola S, Sozzi G, Gasparini G, Menard S, Gatter KC, Harris AL, Fox S, Buyse M, Pilotti S, Pierotti M and Rilke F (1997) Non-small-cell lung carcinoma tumor growth without morphological evidence of neo-angiogenesis. *Am J Pathol* **151**: 1417–1423

Rak J, Mitsuhashi Y, Bayko L, Filmus J, Shirasawa S, Sasazuki T and Kerbel RS (1995) Mutant *ras* oncogenes up-regulate VEGF/VPF expression: implications for induction and inhibition of tumor angiogenesis. *Cancer Res* **55**: 4575–4580

Saclarides TJ, Speziale NJ, Drab E, Szeluga DJ and Rubin DB (1994) Tumor angiogenesis and rectal carcinoma. *Dis Colon Rectum* **37**: 921–926

Skobe M, Rockwell P, Goldstein N, Vosseler S and Fusenig NE (1997) Halting angiogenesis suppresses carcinoma cell invasion. *Nature Med* **11**: 1222–1227

Takahashi Y, Kitadai Y, Bucana CD, Cleary KR and Ellis LM (1995) Expression of vascular endothelial growth factor and its receptor, KDR, correlates with vascularity, metastasis, and proliferation of human colon cancer. *Cancer Res* **55**: 3964–3968

Takahashi Y, Tucker SL, Kitadai Y, Koura AN, Bucana CD, Cleary KR and Ellis LM (1997) Vessel counts and expression of vascular endothelial growth factor as prognostic factors in node-negative colon cancer. *Arch Surg* **132**: 541–546

Takebayashi Y, Akiyama S, Yamada K, Akiba S and Aikou T (1996*a*) Angiogenesis as an unfavorable prognostic factor in human colorectal carcinoma. *Cancer* **78**: 226–231

© Cancer Research Campaign 1999

Takebayashi Y, Akiyama S, Yamada K, Akiba S, Miyadera K, Sumizawa T, Yamada Y, Murata F and Aikou T (1996*b*) Clinicopathological and prognostic significance of an angiogenic factor, thymidine phosphorylase, in human colorectal carcinoma. *J Natl Cancer Inst* **88**: 1110–1117

Tomisaki S, Ohno S, Ichiyoshi Y, Kuwano H, Maehara Y and Sugimachi K (1996) Microvessel quantification and its possible relation with liver metastasis in colorectal cancer. *Cancer* **77**: 1722–1728

Tanigawa N, Amaya H, Matsumura M, Lu C, Kitaoka A, Matsuyama K and Muraoka R (1997) Tumor angiogenesis and mode of metastasis in patients with colorectal cancer. *Cancer Res* **57**: 1043–1046

Van Meir EG, Polverini PJ, Chazin VR, Su Huang H, de Tribolet N and Cavenee WK (1994) Release of an inhibitor of angiogenesis upon induction of wild type p53 expression in glioblastoma cells. *Nature Gen* **8**: 171–176

Vermeulen PB, Verhoeven D, Fierens H, Hubens G, Goovaerts G, Van Marck E, De Bruijn EA, Van Oosterom AT and Dirix LY (1995*a*) Microvessel quantification in primary colorectal carcinoma: an immunohistochemical study. *Br J Cancer* **71**: 340–343

Vermeulen PB, Verhoeven D, Hubens G, Van Marck E, Goovaerts G, Huyghe M, De Bruijn EA, Van Oosterom AT and Dirix LY (1995*b*) Microvessel density, endothelial cell proliferation and tumour cell proliferation in human colorectal adenocarcinomas. *Ann Oncol* **6**: 59–64

Vermeulen PB, Gasparini G, Fox SB, Toi M, Martin L, McCulloch P, Pezzella F, Viale G, Weidner N, Harris AL and Dirix LY (1996*a*) Quantification of angiogenesis in solid human tumours: an international consensus on the methodology and criteria of evaluation. *Eur J Cancer* **32A**: 2474–2484

Vermeulen PB, Roland L, Mertens V, Van Marck E, De Bruijn EA, Van Oosterom AT and Dirix LY (1996*b*) Correlation of intratumoral microvessel density and p53 protein overexpression in human colorectal adenocarcinoma. *Microvasc Res* **51**: 164–174

Vermeulen PB, Libura M, Libura J, O'Neill PJ, Van Dam P, Van Marck E, Van Oosterom AT and Dirix LY (1997) Influence of investigator experience and microscopic field size on microvessel density in node-negative breast carcinoma. *Breast Cancer Res Treat* **42**: 165–172

Weidner N, Semple JP, Welch WR and Folkman J (1991) Tumor angiogenesis and metastasis – correlation in invasive breast carcinoma. *N Engl J Med* **324**: 1–8

Wolmark N, Fisher B and Wieand S (1986) The prognostic value of the modifications of the Dukes' C class of colorectal cancer. *Ann Surg* **203**: 115–121

*© Cancer Research Campaign 1999*
